# Induction of GD3/α1-adrenergic receptor/transglutaminase 2-mediated erythroid differentiation in chronic myelogenous leukemic K562 cells

**DOI:** 10.18632/oncotarget.20080

**Published:** 2017-08-09

**Authors:** Sun-Hyung Ha, Sung-Koo Kang, Hyunju Choi, Choong-Hwan Kwak, Fukushi Abekura, Jun-Young Park, Kyung-Min Kwon, Hyeun-Wook Chang, Young-Choon Lee, Ki-Tae Ha, Bo Kyeng Hou, Tae-Wook Chung, Cheorl-Ho Kim

**Affiliations:** ^1^ Molecular and Cellular Glycobiology Unit, Department of Biological Sciences, SungKyunKwan University, Seoburo, Jangan-Gu, Kyunggi-Do, Korea; ^2^ Research Institute, Davinch-K Co., Ltd., Geumcheon-gu, Seoul, Korea; ^3^ College of Pharmacy, Yeungnam University, Gyeongsan, Korea; ^4^ Faculty of Medicinal Biotechnology, Dong-A University, Busan, Korea; ^5^ Division of Applied Medicine, School of Korean Medicine, Pusan National University, Yangsan City, Gyeongsangnam-Do, Korea; ^6^ Korean Bioinformation Center, Korea Research Institute of Bioscience and Biotechnology, Daejeon, Korea; ^7^ Department of Medical Device Management and Research, Samsung Advanced Institute for Health Sciences and Technology (SAIHST), Sungkyunkwan University, Seoul, Korea

**Keywords:** adrenergic receptor, transglutaminase 2, ganglioside GD3, erythroid differentiation, human chronic myelogenous leukemia K562 cell

## Abstract

The disialic acid-containing glycosphingolipid GD3 recruited membrane transglutaminase 2 (TG2) as a signaling molecule for erythroid differentiation in human chronic myelogenous leukemia (CML) K562 cells. The α1-adrenergic receptor (α1-AR)/TG2-mediated signaling pathway regulated GD3 functions, including gene expression and production, to differentiate CML K562 cells into erythroid lineage cells. Epinephrine, an AR agonist, increased membrane recruitment as well as GTP-photoaffinity of TG2, inducing GD3 synthase gene expression. Epinephrine activated PI3K/Akt signaling and GTPase downstream of TG2 activated Akt. The coupling of TG2 and GD3 production was specifically suppressed by prazosin (α1-AR antagonist), but not by propranolol (β-AR antagonist) or rauwolscine (α2-AR antagonist), indicating α1-AR specificity. Small interfering RNA (siRNA) experiment results indicated that the α1-AR/TG2-mediated signaling pathway activated PKCs α and δ to induce GD3 synthase gene expression. Transcription factors CREB, AP-1, and NF-κB regulated GD3 synthase gene expression during α1-AR-induced differentiation in CML K562 cells. In addition, GD3 synthase gene expression was upregulated in TG2-transfected cells via α1-AR with expression of erythroid lineage markers and benzidine-positive staining. α1-AR/TG2 signaling pathway-directed GD3 production is a crucial step in erythroid differentiation of K562 cells and GD3 interacts with α1-AR/TG2, inducing GD3/α1-AR/TG2-mediated erythroid differentiation. These results suggest that GD3, which acts as a membrane mediator of erythroid differentiation in CML cells, provides a therapeutic avenue for leukemia treatment.

## INTRODUCTION

The adrenergic receptors (ARs) have been known with three subtypes (α1, α2, and β), and α1 AR is further subclassified into 3 subtypes (α1A = C, α1B, and α1D) by parameters of its receptor-ligand interaction and receptor-mediated signaling [1–4]. Naturally-well known ligands of the α1-AR are, for example, catecholamines, epinephrine, and norepinephrine. The α1-AR is involved in glycogenolysis, sympathetic nervous system events, and arteriolar smooth and cardiac muscle contraction; it has been related with the pathogenic cardiac arrhythmias and hypertrophy [1, 5]. ARs are also known to couple with the ubiquitous transglutaminase-2 (TG2) [6], and are thus involved in TG signaling. TG2 has been known to have two enzymatic properties of 1) Ca^2+^-dependent transamidase that cross-links proteins or incorporates polyamines into proteins [7, 8, 2] GTPase (Gh) that functions as a receptor signaling GTP-binding protein [9]. GTP-bound TG2 mediates signal transductions of membrane receptor to intracellular effectors. The studied example of this phenomenon is the α1-AR-down-streamed enhancement of phospholipase C activity [10, 11], in which the α1-AR activates GTP binding capacity of TG2. Phospholipase C up-regulated by GTP-bound TG2 consequently stimulates phosphoinositide lipid hydrolysis. As the TG2-coupled receptors, the α1B-AR [12], α1D-AR [12], α-thromboxane [13], and oxytocin [14] receptors are known to date. TG2 is mainly localized in the cytosol, while a small portion of the TG2 fraction is attached to the membrane. The externalization of the TG2 from cells is stringently controlled to stabilize the extracellular matrix and facilitate cellular behaviors such as adhesion, migration and motility [15]. For example, an integrin-binding ability of TG2 leads to adhesion and migration on fibronectin [16]. A previous study performed by our group suggested that TG2 overexpression as well as membrane recruitment and membrane localization of TG2 accelerate erythroid differentiation of K562 cells [17].

**Figure 1:**
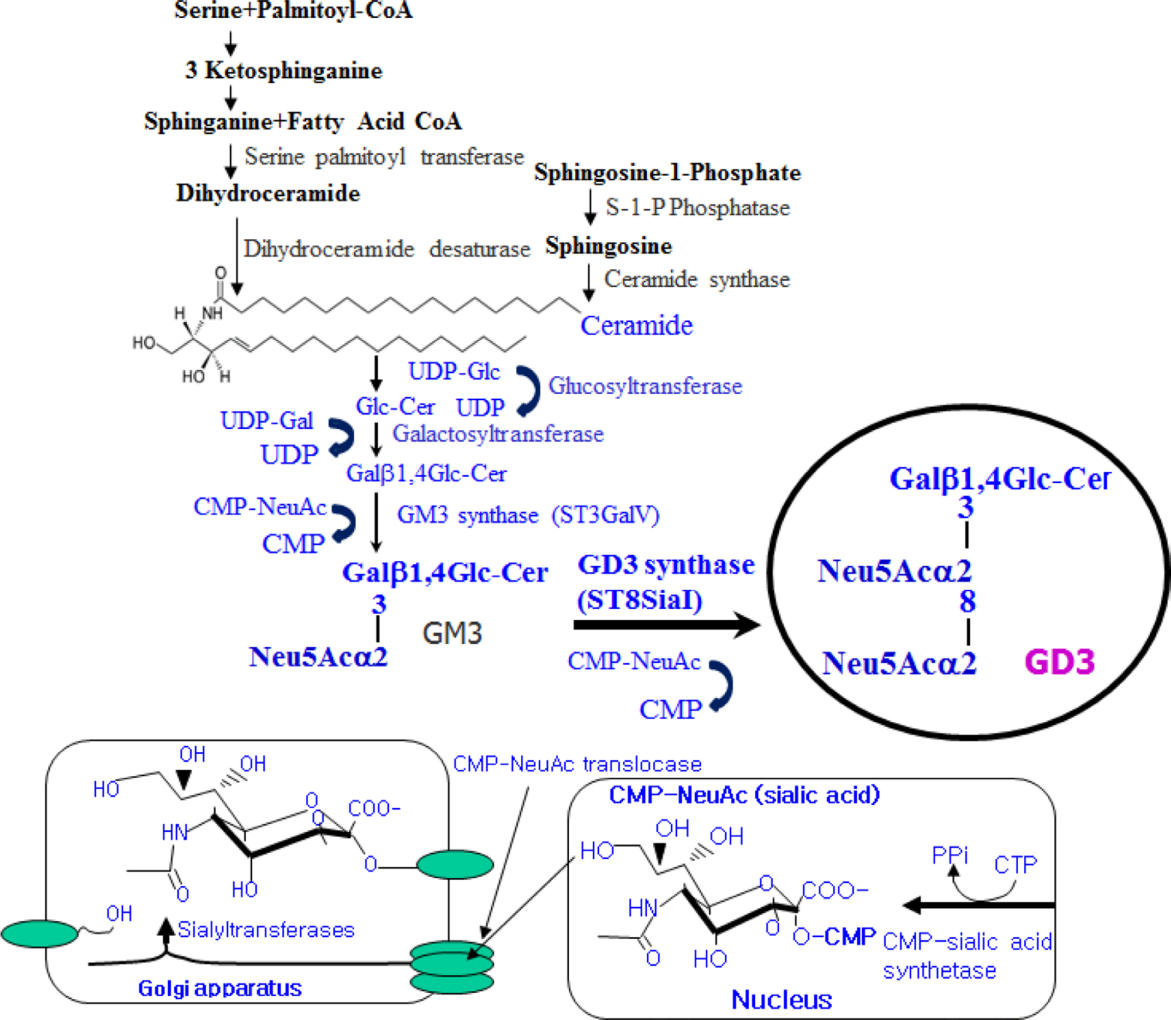
Schematic illustration of disialyl ganglioside GD3 synthesis in mammalian cells

Mammalian cells are surfaced with carbohydrates as forms of glycoproteins, glycolipids and glycosaminoglycans. The expression of distinct carbohydrates is restricted to specific cell types. Among glycolipid components, gangliosides are sialylated glycosphingolipids and ubiquitously expressed components of the outer surface of vertebrate cells [18]. Changes in ganglioside metabolism, distribution and composition are associated with growth, differentiation, adhesion, migration, motility, immune response, signal transduction, receptor signaling, tumorigenesis, and angiogenesis [19]. For example, a surface shift in accumulation of specific gangliosides, pathways of ganglioside biosynthesis, cellular ganglioside patterns and formation of new types of ganglioside is pivotal in the cellular differentiation [20]. Therefore, the formation of specific gangliosides on the cellular membrane may function as a key regulator in the induction of cellular differentiation and as a specific determinant of the differentiation direction. However, the molecular mechanism(s) underlying that gangliosides regulate cellular differentiation is still poorly understood. Disialoganglioside GD3 synthase (ST8Sia I/SAT II) known as CMP-NeuAc:GM3 α2,8-sialyltransferase converts monosialyl form GM3 into disialyl form GD3, as shown in Figure 1. GD3, the product of GD3 synthase, is expressed predominantly in cells and tissues of the central nervous system [21] and melanoma cells [22]. Its expression increases during development and cellular differentiation, indicating that GD3 is a key mediator of cell differentiation [23, 24, 25]. These diverse effects indicate that tightly regulated mechanisms control GD3 function by affecting its intracellular levels, localization, structure, and expression.

Previously, our group demonstrated that GD3 plays a critical role in membrane recruitment of transglutaminase to accelerate erythroid differentiation of chronic myelogenous leukemia (CML) K562 cells (Ref., 26, Kang *et al*., Proteomics 8, 3317-3328, 2008). In the present study, we demonstrate that up-regulation of GD3 synthase is directly coupled with TG2 and that TG2 is serially coupled with the ARs for erythroid differentiation of CML K562 cells. The directly coupled α1-AR/TG complex regulates GD3 expression through activation of PKCs α and δ to induce erythroid differentiation of CML K562 cells. Given the current findings, a novel mechanism for action of ganglioside GD3 in CML treatment is proposed. Although in leukemia treatment, the first molecularly targeted drug, Imatinib, effective for CML, is remarkably successful, the resistance emergence to the Imatinib has reduced the therapeutic anticipation for this CML type of leukemia, necessitating that an agent which can provide a definitive cure for CML should be developed in the near future. Therefore, the present study might provide a rationale for combination therapy consisting of AR agonists, hemic/tRA, GD3, and Imatinib or other pharmacological agents.

## RESULTS

### Recruitment of TG2 into the membrane mediated by epinephrine, an AR agonist, in chronic myelogenous leukemic (CML) K562 cells

**Figure 2:**
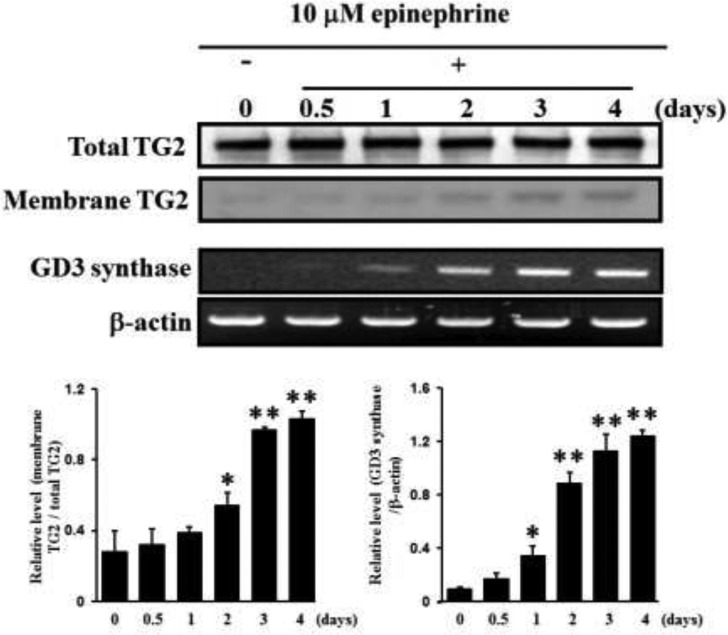
Increase in membrane recruitment of TG2 in response to epinephrine First, 50 μg of membrane proteins were isolated and subjected to 10% SDS-PAGE, as described in Experimental Procedures. TG2 expression levels were determined by Western blot analysis. RT-PCR analysis of GD3 synthase mRNA. Total RNAs were isolated from K562 cells after 0, 0.5, 1, 2, 3, or 4 days of treatment with 10 μM epinephrine. Then 1 μg of total RNA from each cell was subjected to RT-PCR. β-Actin indicates that equal amounts of RNA were loaded in each lane. Data are representative of three experiments (means ± SD). *P < 0.05 vs. control (0).

In our previous papers [22, 27], the membrane-bound molecule TG2 was shown to be involved in erythroid differentiation of CML K562 cells. In this study, the localization of TG2 was examined with and without 10 μM epinephrine, an AR agonist. Membrane-localized TG2 was isolated from the cytosol fraction and Western blot was analyzed. The amount of membrane-bound TG2 in the cells treated with 10 μM epinephrine gradually increased time-dependently in response to epinephrine (Figure 2). To investigate whether epinephrine, an AR agonist, enhance the GD3 synthase gene expression to recruit TG2 to membrane region in K562 cells, RT-PCR analysis was carried out using the GD3 synthase gene-specific primers. As shown in Figure 2, the results of RT-PCR analysis showed that the induction of GD3 synthase mRNA has been detected within 12 hours after treatment with 10 μM epinephrine, time-dependently increasing.

### Akt activation and inactivation of ERK1/2 phosphorylation by epinephrine in K562 cells

**Figure 3:**
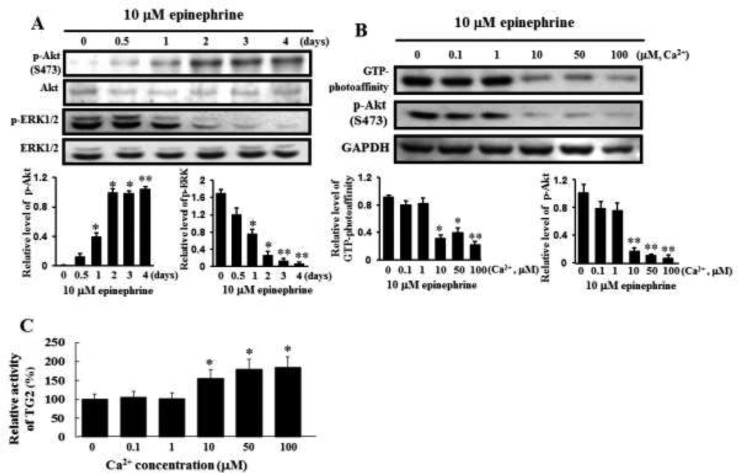
Activation of Akt phosphorylation and inactivation of ERK1/2 by treatment with 10 μM epinephrine and comparison of the GTP photoaffinity of membrane-bound TG2 and its transglutaminase activity at varying calcium concentrations (**A**) Activation of Akt phosphorylation and inactivation of ERK1/2 following treatment with 10 μM epinephrine. Cells were incubated with 10 μM epinephrine for the time periods indicated in the figure. Then, 25 μg of protein was subjected to 10% SDS-PAGE. Phospho-specific antibodies were used to measure the activation of Akt and ERK1/2. The blots were stripped and then reprobed with anti-Akt and anti-ERK1/2 antibodies. Data are representative of three experiments. (**B**) Influence of GTP-bound TG2 on Akt phosphorylation following incubation with 10 μM epinephrine for 2 days at different Ca2+ concentrations. The GTP binding activity of membrane-bound TG2 was determined using affinity labeling with radioactive GTP, as described in Experimental Procedures. [α-^32^P]GTP-bound TG2 was immunoprecipitated with anti-TG2 antibody. The bound radiolabeled GTP was visualized by autoradiography, following 10% SDS-PAGE. GAPDH indicated that an equal amount of proteins was loaded in each lane (lower panel). Data are representative of three experiments. (**C**) TGase activities in the cytosolic fraction were measured by incubation with 10 μM epinephrine for 2 days at different Ca^2+^ concentrations. Data are representative of three experiments (means ± SD). *P < 0.05 and **P < 0.01, vs. control (0).

It has been known that G-protein-coupled receptors phosphorylate Akt during cellular responses [27]. The capability of epinephrine to activate Akt signaling was then examined using specific antibodies to react the phosphorylated form of Akt. Akt phosphorylation increased in a time-dependent manner following application of 10 μM epinephrine, while phosphorylation of ERK1/2 was down-regulated by 10 μM epinephrine (Figure 3A). To determine whether calcium is responsible for Akt phosphorylation, levels of membrane-bound TG2 and Akt phosphorylation were determined at several calcium concentrations. GTP photoaffinity of membrane TG2 and Akt phosphorylation increased at low calcium levels in response to 10 μM epinephrine (Figure 3B). Cytosolic TG enzyme activity increased at high calcium levels (Figure 3C), suggesting that the TG activity might turn over at high calcium levels and the GTPase function of TG2 might primarily influence Akt phosphorylation in CML K562 cells.

### α1-AR-mediated membrane recruitment of TG2 and increase in GD3 synthase gene expression in K562 cells

**Figure 4:**
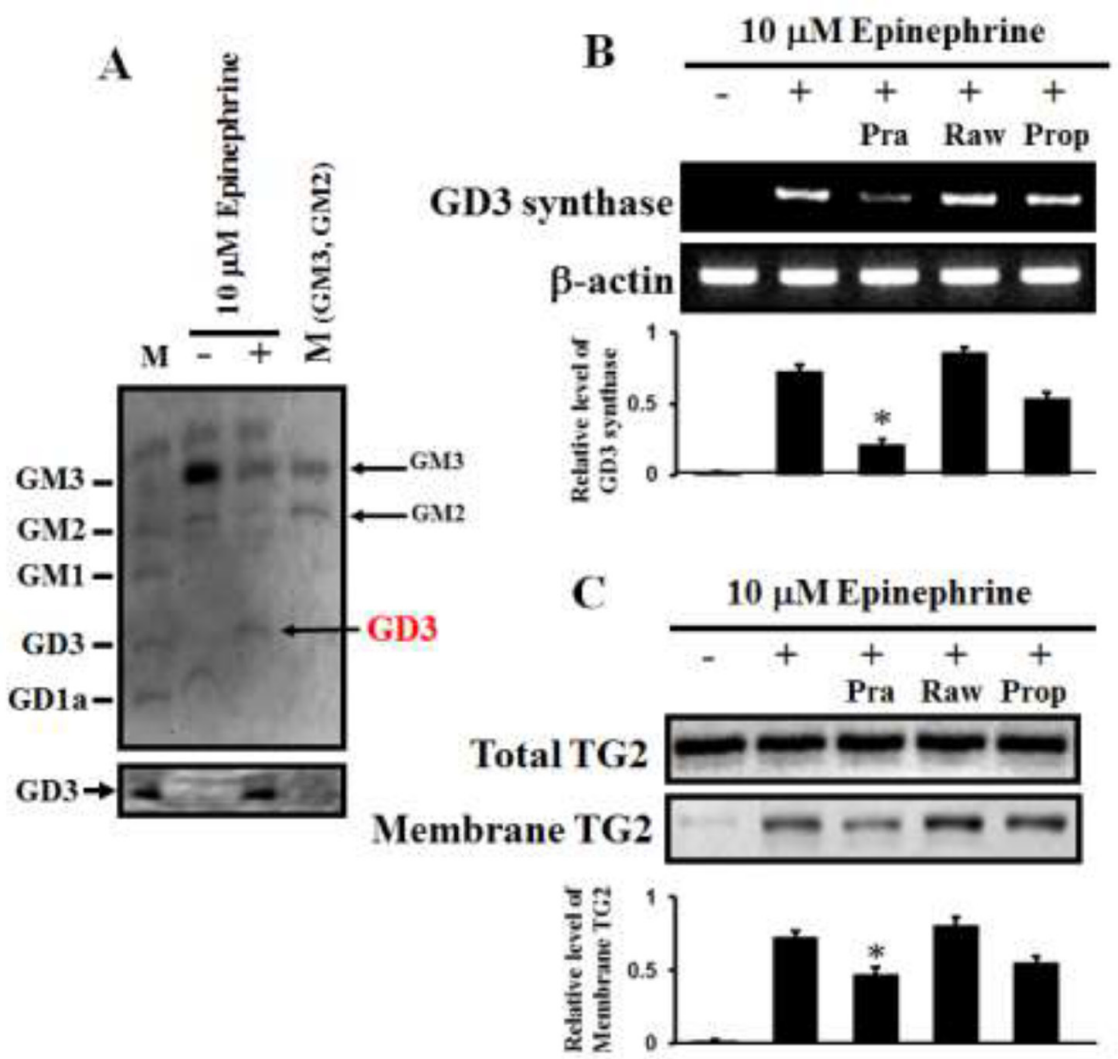
α1-AR-mediated increases in GD3 expression and membrane recruitment of TG2 (**A**) Ganglioside profiles. Gangliosides were isolated from 1 × 10^9^ K562 cells. Separated gangliosides were revealed by spraying with the resorcinol hydrochloride reagent (upper panel). Immunostaining of GD3 with a GD3-specific antibody (Lower panel). (**B**) α1-AR-specific GD3 synthase expression as measured by RT-PCR. The cells were incubated for 2 days with 10 μM epinephrine in the presence of the combination containing the AR antagonists prazosin (α1), rauwolscine (α2), and propranolol (β). (**C**) Levels of membrane-bound TG2 in response to epinephrine in the presence of several AR antagonists. Total TG2 level indicates that equal amounts of protein were loaded. M (marker) denotes a series of gangliosides (GM3, GM2, GM1, GD3, and GD1a). Data are representative of three experiments (means ± SD). *P < 0.05 vs. control (0).

When the ganglioside patterns seen on HPTLC and immunostaining with GD3 antibody were compared (Figure 4A), the expression of the ganglioside GD3 was clearly increased in K562 cells treated with 10 μM epinephrine. However, the levels of GM3 and GD1a were diminished, as measured on HPTLC. To investigate which ARs are involved in GD3 synthase expression, the cells were treated with 15 μM prazosin, 2 μM rauwolscine, and 10 μM propranolol, which are α1, α2, and β-adrenergic antagonists, respectively. When the GD3 synthase gene expression and membrane-bound TG2 levels were examined by RT-PCR and immunoblot analysis, 15 μM prazosin only reduced the levels of GD3 synthase gene expression and membrane TG2. As shown in Figure 4B, GD3 synthase gene expression was down-regulated when the cells were treated with prazosin in the presence of 10 μM epinephrine, but no changes were detected when the cells were treated with rauwolscine or propranolol, indicating that the reaction is α1-AR specific. Also, membrane-bound TG2 levels were decreased only in the presence of prazosin (Figure 4C).

### GD3 synthase gene expression is regulated by PKC α as well as by PKC δ in K562 cells

**Figure 5:**
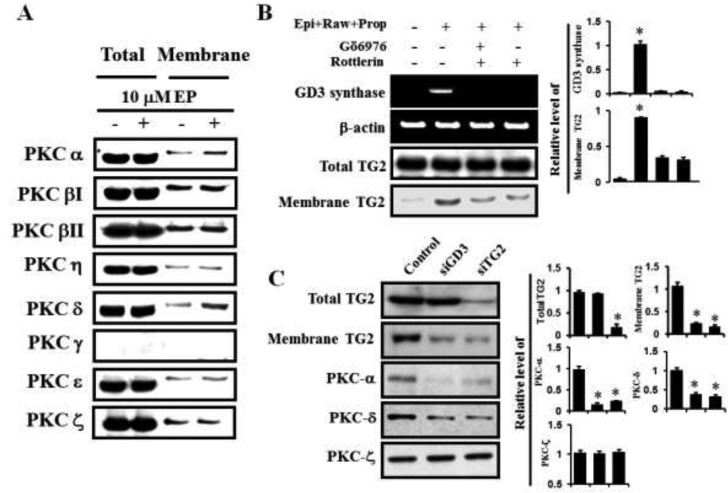
PKC α and δ mediated GD3 synthase expression via α1-AR/TG2 K562 cells were incubated with 10 μM epinephrine in the presence of the combination containing rauwolscine and propranolol for 2 days. (**A**) Membrane translocation of PKC α and PKC δ by α1-AR. (**B**) Down-regulation of GD3 synthase expression and membrane recruitment of TG2 following treatment with the PKC α and PKC δ inhibitors, Gö6976 and Rottlerin, respectively. (**C**) Inhibitory effect of TG2 and GD3 synthase siRNAs on the activation of PKC α and PKC δ by α1-AR. Data are representative of three experiments (means ± SD). *P < 0.05 vs. control (0).

From the above results regarding the prazosin, rauwolscine, and propranolol treatments, it was evident that the α1-AR-mediated signaling pathway regulates the GD3 synthase gene expression and membrane recruitment of TG2 in order to differentiate K562 cells into the erythroid lineage. To address whether the PKC pathway is involved in α1-AR/TG2 downstream signaling, Western blot analysis was performed. Membrane translocation levels of PKC α and δ were increased in response to 10 μM epinephrine (Figure 5A). To further investigate the GD3 synthase and TG2 behaviors, the cells were co-treated with α2 and β antagonists (rauwolscine and propranolol, respectively) only, but not with α1 antagonist prazosin. Based on these results, we treated the cells with PKC α and PKC δ inhibitors, Gö6976 and Rottlerin, respectively. GD3 synthase gene expression was apparently abolished by these inhibitors, as demonstrated by RT-PCR (Figure 5B, upper panel), which is consistent with TG2 membrane recruitment (Figure 5B, lower panel). siRNA analysis of GD3 synthase and TG2 also showed that membrane translocation of PKCs α and δ decreased in response to epinephrine (Figure 5C). However, membrane translocation of PKC ζ was not observed.

### Promoter assay of GD3 synthase gene expression and EMSA analysis in K562 cells

**Figure 6:**
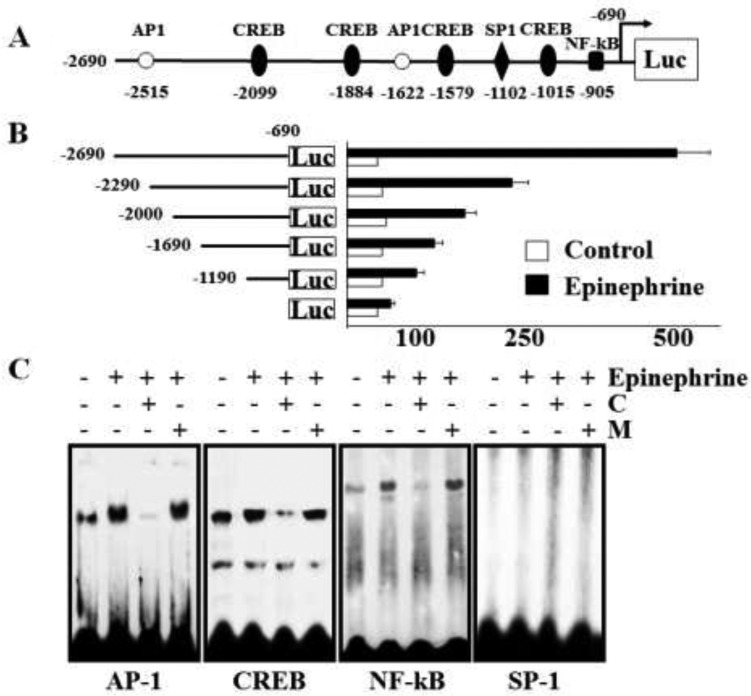
Promoter assay of GD3 synthase and EMSA analysis Cells were incubated with 10 μM epinephrine and rauwolscine or propranolol. (**A**) The promoter region of GD3 synthase. The region from −2690 to −690 contains two AP-1 (consensus sequence motif: 5′-TGACG-3′), four CREB (5′-TGACGTCA-3′), one SP-1 (5′-GGGTGG-3′), and one NF-κB (5′-GGGAGACCT-3′) putative regulatory elements. (**B**) Analysis of deletion mutants of the GD3 synthase gene promoter in K562 cells in response to 10 μM epinephrine. pGL3-basic (without any promoter or enhancer) was used as a negative control. pGL3-control (with the SV40 promoter and enhancer) was used as a positive control. Each construct was co-transfected into K562 cells with pCMV as the internal control. The transfected cells were incubated in the presence (solid bars) or absence (open bars) of 10 μM epinephrine for 2 days. Relative luciferase activity was normalized to β-galactosidase activity derived from pCMV. (**C**) Electrophoretic mobility shift assay (EMSA). Nuclear protein fractions were isolated after incubation with or without 10 μM ephinephrine for 2 days. DNA–protein complexes were analyzed on a 4% non-denaturing polyacrylamide gel. For competition experiments, a 50-fold molar excess of unlabeled wild-type oligonucleotides (C) or unlabeled mutant oligonucleotides (M) was used. The results were obtained from three independent experiments.

To characterize the promoter region regulating the transcriptional activity of the GD3 synthase gene during stimulation of K562 cells with epinephrine, we constructed a reporter plasmid (pGL) linking the luciferase gene and 5′-flanking regions (−2690–−690) of the GD3 synthase gene based on our previous report [28]. The constructed reporter plasmid (or empty pGL3 plasmids as a control) was transfected into K562 cells and regulation of the GD3 synthase promoter activity by 10 μM epinephrine was examined. The region from −2690 to −690 bp contains putative binding sites located within the 5′-flanking GD3 sequence that include perfect matches with two AP-1 (consensus sequence motif: 5′-TGACG-3′), four CREB (5′-TGACGTCA-3′), one SP-1 (5′-GGGTGG-3′) and one NF-κB (5′-GGGAGACCT-3′) putative regulatory elements (Figure 6A). As shown in Figure 6B, −2690 - 690/pGL3 resulted in about five-fold higher promoter activity than vector alone. To further analyze the epinephrine-responsive region regulating the transcriptional activity of GD3 synthase in K562 cells, 5′-deleted GD3 synthase promoters of various sizes (−2290 - −690/pGL3 to −1190 - −690/pGL3) were constructed and transfected them into K562 cells. Deletion of nucleotides −2690 - −1190 resulted in attenuated transcriptional activity. However, promoter activity did not reach basal levels in the deletion mutants. Based on these results, in order to determine whether these binding sites contribute to transcriptional regulation of GD3 synthase in epinephrine-treated K562 cells, EMSA analysis was performed using K562 nuclear extracts in the presence or absence of epinephrine. To further characterize the binding sites for AP-1, CREB, SP-1, and NF-κB motifs located within the −269 ∼ −1190 bp region of the GD3 synthase promoter, four synthetic double-stranded oligonucleotides were ^32^P-labeled and subjected to EMSA (Figure 6C). The EMSA results clearly showed increased complex formation with AP-1, CREB, and NF-κB probes, but not with SP-1 probe. The results clearly suggest that transcription factors AP-1, CREB, and NF-κB activate the GD3 synthase gene upon stimulation with epinephrine. However, potential positive regulatory elements including the AP-1 site (−promoter region, −2690 to −2290 bp) regulate the GD3 synthase gene induced by epinephrine in K562 cells although there are transcriptional activities of several transcription factors.

### α1-adrenergic receptor/TG2-mediated GD3 synthase expression and erythroid differentiation marker genes in K562 cells

**Figure 7:**
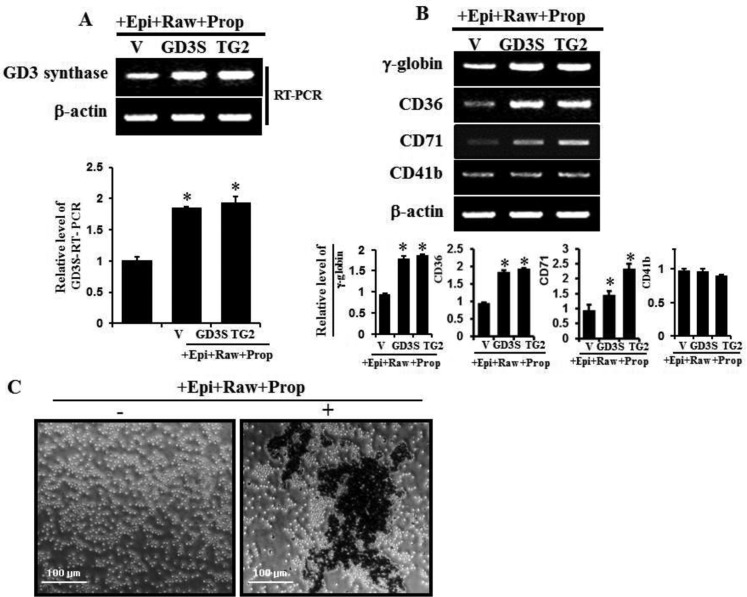
GD3 synthase expression and expression of several erythroid differentiation marker genes induced by α1-AR/TG2-mediated signaling K562 cells were transfected with GD3 synthase (GD3S) and TG2 cDNAs. Without α1-AR antagonist prazosin, cells were treated with α2-AR and β-AR-specific antagonists such as rauwolscine and propranolol in the presence of 10 μM epinephrine, classified as +Epi+Raw+Prop. Then, 1 μg of total RNA isolated from K562 cells was subjected to RT-PCR using primers specifically designed for megakaryotic or several erythroid lineage marker genes, as described in Experimental Procedures. β-Actin mRNA expression indicated that equal amounts of mRNA were used for RT-PCR in each lane. (**A**) RT-PCR of GD3 expression induced by α1-AR/TG2-mediated signaling. (**B**) Induction of several differentiation marker genes by α1-AR/TG2-mediated signaling. (**C**) Cells were incubated with 10 μM epinephrine for 2 days and benzidine staining was performed as described in the ‘Materials and Methods’ section. Data are representative of three experiments (means ± SD). *P < 0.05 vs. control (0).

To investigate the expression levels of α1-AR/TG2-mediated GD3 synthase and erythroid differentiation marker genes, cells were transfected with a vector, GD3 synthase cDNA and TG2 cDNA. The transfectant cells were treated with 2 μM rauwolscine and 10 μM propranolol, as α2 and β-adrenergic antagonists, respectively, whereas cells were not treated with prazosin, an α1-adrenergic antagonist, because prazosin suppresses epinephrine-induced GD3 synthase gene expression and TG2 membrane recruitment, as shown in Figure 4C and D. GD3 synthase gene expression clearly increased in TG2-transfected cells in response to the condition of 10 μM epinephrine+2 μM rauwolscine and 10 μM propranolol (+Epi+Raw+Prop), as evidenced by RT-PCR (Figure 7A). To characterize differentiation in further detail, erythroid differentiation was also confirmed by RT-PCR using specific markers of γ-globin and CD36 genes after incubation for 48 hr under the same condition (+Epi+Raw+Prop). As shown in Figure 7B, γ-globin and CD36 mRNA levels increased in GD3 synthase- and TG2 gene-transfected cells. We further assessed whether the expression of CD71 gene was increased in K562 cells because CD71, transferrin receptor, is one of the crucial erythroid markers and is also involved in iron delivery into the cells. When the transfected K562 cells were exposed to the condition of +Epi+Raw+Prop, the CD71 gene expression was increased (Figure 7B). On the other hand, the expression of CD41b, a megakaryotic differentiation marker gene, did not change, as was expected. From the results, we suggest that erythroid differentiation of K562 cells involves collaboration with membrane-bound TG2 recruited by GD3 following epinephrine treatment under the same condition (+Epi+Raw+Prop). This speculation is supported by benzidine dye staining on phase contrast microscopy (Figure 7C).

## DISCUSSION

Our study provides new insights into the regulatory mechanisms that underlie epinephrine-induced ganglioside GD3 gene expression during erythroid differentiation of K562 cells, somewhat elucidating the strong relationship between GD3 and leukemic cell differentiation. Our initial aim was to determine whether membrane recruitment of TG2 occurs in response to hemin/all-trans retinoic acid (tRA), and to study whether expression of GD3 induces membrane recruitment of TG2 along with erythroid differentiation [26].

The ganglioside profile of the K562 cell line depends upon both differentiation stage and differentiation direction [29]. It was previously known that GM2, GM3, and GD1a are the mainly expressed gangliosides in K562 cells, as reported by Suzuki *et al*. [30]; we confirmed their results in this study via resorcinol staining. It has been previously reported that GM3 is a major component of the ganglioside profile of human platelets [31]. Nakamura *et al.* [32] reported that GM3 levels increase during TPA-induced megakaryocytic differentiation and decrease during hemin-induced erythroid differentiation. This suggests that the megakaryotic lineage is linked with GM3-enriched ganglioside. In the present study, GM3 levels were decreased in epinephrine-treated cells, suggesting that GM3 content is a glycolipid indicator of bi-directional differentiation of cells. However, an increase in GM3 levels may not be sufficient to induce erythroid differentiation of K562 cells.

On the other hand, it is known that the Bcr-Abl protein exhibits an escalated tyrosine kinase activity in chronic myelogenous leukemia models such as K562 cells, and it activates various phosphorylation pathways [33, 34]. To examine whether Bcr-Abl phosphorylates Akt, proteins in vector- and GD3 synthase-transfected cells were also immunologically reacted with anti-c-Abl antibody and immunoblotted with phospho-tyrosine antibody. As expected, no any difference of phosphorylation level was observed in the tyrosine phosphorylation of Bcr-Abl in response to epinephrine. This suggests that adrenergic receptor-related Akt phosphorylation is not related with the Bcr-Abl signaling pathway. To assess the effects of α1-AR, α2-AR and β-AR-specific antagonists on membrane recruitment of TG2 and GD3 synthase gene expression in K562 cells, cells were treated with AR-specific drugs such as prazosin, rauwolscine, and propranolol in the presence of 10 μM epinephrine. GD3 synthase gene expression and TG2 membrane recruitment were specifically suppressed by prazosin, indicating the role of the α1-AR-mediated signaling pathway in K562 cells. Then, we speculated that the α1-adrenergic receptor-mediated pathway leads to the activation of transcriptional regulators that enhance transcription of the GD3 synthase gene. The results of luciferase assays and EMSA showed that the activity of AP-1 is mainly required for the expression of GD3 synthase, which is consistent with previous observations which show that AP-1 is activated by PKC [35] as well as by PI3K [36]. It has been reported that inhibition of ERK is associated with erythroid differentiation [37, 38]. Our results also showed that ERK phosphorylation was inhibited by epinephrine treatment. One possible explanation for the inhibition of ERK1/2 by epinephrine is that epinephrine increases the GTP binding capacity of TG2, which may compete with the small GTP binding protein Ras; Ras is required for ERK activation [39]. Antonyak *et al.* [40] have shown that activation of the Ras-ERK pathway suppresses the RA-induced GTP binding capacity of TG2 as well as its expression. Therefore, changes in the GTP binding capacity of membrane-bound TG2 induced by epinephrine might be responsible for inhibition of ERK1/2 phosphorylation. This issue requires further investigation. CREB, an Akt substrate, is known as a transcription factor that affects genes involved in erythroid differentiation [17] and its phosphorylation is mediated by α1-AR [41]. NF-κB may be activated in response to epinephrine, raising the possibility that PKC activation as well as PI3K activity is required for adrenergic receptor-mediated GD3 synthase expression. This possibility is supported by our observation that promoter activities of mutant constructs with GD3 synthase promoter region deletions appeared similar to those of controls in the luciferase assay. Although there is an SP 1-binding site in the GD3 synthase promoter region [28], we could not detect any activation of SP-1.

The position of TG2 in the GTPase cycle (as a TGase) remains unclear. TGase activation and deactivation are reported to be controlled by two cofactors [8] such as the activator Ca^2+^ and the inhibitor GTP. Calcium effectively inhibits the GTP binding to TG2 [42]. Ca^2+^ is normally stored in the endoplasmic reticulum and the sarcoplasmic reticulum as intracellular organelles [43]. Although the low concentration (ranged between 100–200 nM) of calcium is stored in cytosolic fraction, its cytosolic concentration rapidly increases up to ≤ 1–10 μM when Ca^2+^ is released from intracellular stores. In the case of GTP, the cellular concentration is normally higher than that of GDP in the most cells [44], and at physiological concentrations of GTP, TG displayed no TGase activity even in the concentration of 10 μM Ca^2+^ in permeabilized cells [45]. However, at higher 100 μM level of Ca^2+^ and low concentrations of nucleotides, TG2 displayed TGase activity. Our previous study which showed that GTP photoaffinity of membrane-bound TG2 was increased [17] and the present result which showed that GTP photoaffinity of membrane-bound TG2 was higher at lower calcium concentrations support this result. In addition, at low calcium levels, Akt phosphorylation was induced, suggesting that TGase function is low under normal and physiological condition of cells

Protein kinase C (PKC) is involved in cross-talk between many signaling pathways and is activated by various different processes [46]. For both cPKC (conventional PKCs: α, β, and γ) and nPKC (novel PKCs: δ, ε, η, and θ) isoforms, the enzymatic activations are generally regulated by their translocation to cellular membranes, allowing protein interaction [47]. In this regard, we investigated downstream signaling of AR/TG2. GD3 synthase gene expression and membrane recruitment of TG2 were elevated by α1-AR. However, this phenomenon was blocked by PKC inhibitors, indicating that the α1-AR/TG2 mediated signaling pathway may primarily activate PKC, and then, PKC induces the phosphorylation of several ARs [46, 48]. Recently, Marchisio *et al*. found that activation of PKCs α, δ, and ζ plays a crucial role in the dealing with erythroid cell differentiation [49]. It was shown that expression of wild-type TG2 and activity-lacking mutant TG2 significantly increased in the rate of peak Ca^2+^ levels and a prolonged increase in cytosolic Ca^2+^ upon activation of the α1B-AR [10, 11], which is consistent with our result that activation of PKC α by α1-AR/TG2 induced GD3 synthase gene expression accompanying membrane recruitment of TG2. However, it is not likely that GD3 is involved in membrane translocation of PKCs, because the results of siGD3 synthase experiments as well as siTG2 experiments showed a decrease in PKC membrane translocation. Moreover, it is interesting to note that α1-AR/TG2 activates PKC δ. Although PKC δ has potentially been function as a general apoptotic mediator, depending on many stimuli, its mechanism is not precisely explained due to the cell type specificity and stimulant nature [50]. For example, PKC δ has previously been reported to slow cell growth rate, induce cell cycle arrest, and stimulate differentiation of various undifferentiated cells [51, 52].

**Figure 8:**
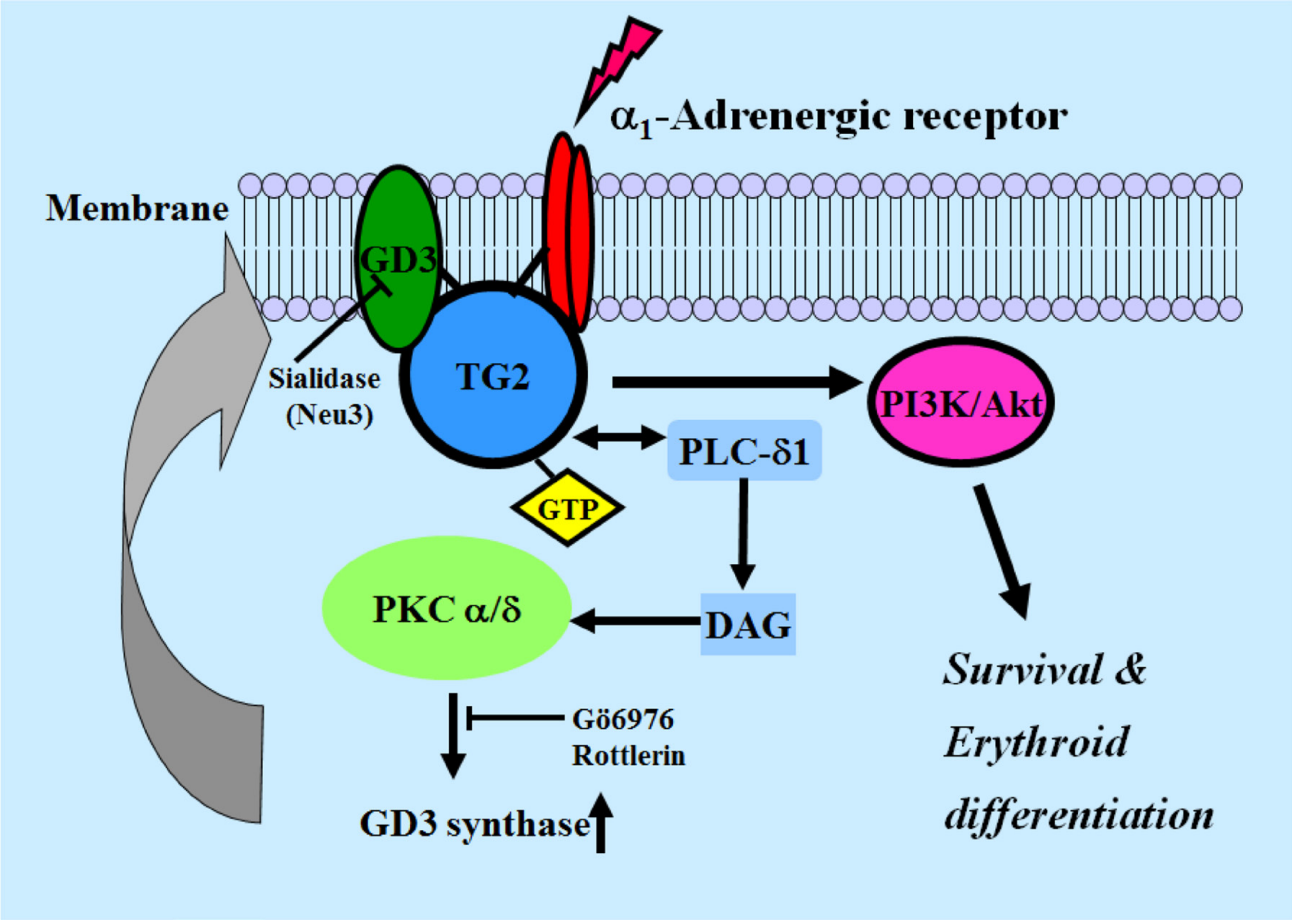
Schematic diagram of GD3 synthase expression induced by α1-AR/TG2-mediated signaling in K562 cells PLC, phospholipase C; PKC, protein kinase C; DAG, diacylglycerol.

In conclusion, the present study suggests a novel idea that membrane recruitment of TG2 by ganglioside GD3 through the α1-AR-mediated signaling pathway (Figure 8) might be a pivotal factor in erythroid differentiation of K562 cells. Together with bone marrow transplantation, a definitive strategy for leukemic therapy is yet needed to develop cure agents. Although the first molecularly targeted Imatinib designed and developed for CML has been evaluated with an outstanding success, the Imatinib-resistance issue reduces its potential to cure this type of leukemia [53, 54]. Some investigators are trying to develop new therapeutic drugs to overcome Imatinib resistance [55, 56]. Our study provides a rationale for combination therapy with AR agonists, hemic/tRA, GD3, and Imatinib or other pharmacological compounds. In summary, we found for the first time that GD3/α1-AR/TG2 mediated leukemic cell differentiation into erythroid lineage, thus confirming that GD3, which acts as an inducer of erythroid differentiation in CML cells, might provide an avenue for leukemia treatment.

## MATERIALS AND METHODS

### Cell culture

The human CML K562 cell line was used for general experiments and stable transfection of GD3 synthase cDNA using the LipofectAMINE reagent according to the supplier's instructions. The 1070 bp cDNA encoding the open reading frame for GD3 synthase gene was inserted into *Hind*III/*Xho*I sites of the pcDNA3 expression vector. TG2 cDNA as a generous gift from Dr. M.J. Im (Chunbuk University, Chunju, Korea) has been used. Cell lines were cultivated and maintained in RPMI-1640 medium, which was supplemented with 10% fetal bovine serum (FBS), 100 μg/ml streptomycin, and 100 U/ml penicillin G (Life Technologies) at 37°C in a humidified 5% CO_2_ incubator. Each cDNA-transfected cells was selected in a cell culture medium containing 600 μg/ml of G418 sulfate and maintained. All experiments were independently performed at least twice using different preparations.

### Reverse transcription-polymerase chain reaction (RT-PCR)

Using the Trizol reagent (Invitrogen, Life Technologies, USA), total RNAs were isolated and prepared from untreated K562 cells as controls and epinephrine-treated K562 cells, respectively. For RT-PCR, one μg of RNA was subjected to reverse transcription with random nanomers utilizing the Takara RNA PCR kit (Takara Shuzo, Shiba, Japan), as recommended by the manufacturer's protocol. cDNA fragments were amplified by PCR with the primers listed in Table 1.

**Table 1:** Primer sequences

Genes	Primer sequences
GD3 synthase (413 bp)	5′- CCCTGCCATTCTGGGTACGAC-3′ (sense)
	5′- CACGATCAATGCCTCCACTGAGATC-3′ (antisense)
Neu3 (371bp)	5′- TACAGTAGAATGTGAAGTGGCAGAG-3′ (sense)
	5′- AGATACCTAGGTCAACCCTCTGTTT-3′ (antisense)
Glycophorin A (416 bp)	5′- GTCAGCAATTGTGAGCATATCAGCA-3′ (sense)
	5′-GATCACTTGTCTCTGCATTTTCTAT-3′ (antisense)
γ-Globin (423 bp)	5′-GGACAAGGCTACTATCACAA-3′ (sense)
	5′-CAGTGGTATCTGGAGGACAG-3′ (antisense)
CD36 (266 bp)	5′-CTGGCTGTGTTTGGAGGTATTCT-3′ (sense)
	5′-AGCGTCCTGGGTTACATTTTCC-3′ (antisense)
CD41b (379 bp)	5′-AGGCCTCTGTCCAGCTAC-3′ (sense)
	5′-GCCATTCCAGCCTCCGTG-3′ (antisense)
CD71 (145 bp)	5′-GCATGTCCGGTTTGCACAT-3′ (sense)
	5′-GACGATCACAGCAATAGTCCC-3′ (antisense)
β-Actin (247 bp)	5′-CAAGAGATGGCCACGGCTGCT-3′ (sense)
	5′-TCCTTCTGCATCCTGTCGGCA-3′ (antisense)

### Preparation of membrane proteins and western blot analysis

Membrane proteins were isolated and Western blot analysis was carried out as described previously [17]. The protein contents of the samples were determined by the Bradford protein determination method using the Bio-Rad protein assay reagent. The following antibodies were used: Anti-phospho-Akt (Ser473), anti-phospho-ERK1/2, and anti-PKCs (SantaCruz, USA), anti-TG2 (CUB 7402, Biomeda, USA), and anti-GAPDH (Chemicon, USA). Immunoblots were visualized and revealed by autoradiography using the enhanced chemiluminescence ECM detection kit (Amersham Biosciences, UK). Anti-α1 adrenergic receptor antibody [EPR11821(2)] (ab192614) was used, as supplied from Abcam Co. (Cambridge, MA, USA).

### Analysis of gangliosides

Isolation and preparation of gangliosides have previously been described by Zeng et al. [57]. The isolated gangliosides were analyzed and visualized by HPTLC separation and detection using Silica Gel 60 plates (Merck, Darmstadt, Germany) as gangliosides were visualized by spraying plates with the resorcinol hydrochoride reagent. Standard gangliosides were commercially purchased from Seikagaku Kougyo CO. (Kyoto, Japan) or Wako Pure Chemicals Co. (Tokyo, Japan). HPTLC plates containing the separated gangliosides were coated with an n-hexane solution containing 0.1% polyisobutylmethacrylate for 1 min and completely dried. The dried plates were then incubated in PBS containing 1% bovine serum albumin (1% BSA/PBS) for 1 h at room temperature (RT), and then the plates were incubated in 1% BSA/PBS containing anti-GD3 antibody (Seikagaku Kougyo Co., Japan) at RT for 2 h. The plates were washed three times with PBS containing 0.05% Tween 20 (PBS-T), and incubated in an HRP-conjugated goat anti-mouse IgM (Biomeda, USA) solution in 1% BSA/PBS for 1 hr at RT. After washing the plates with PBS-T, chemiluminescent reagents (Chemiluminescence Reagent Kit, Amersham Biosciences, UK) were added and the results were visualized by exposure to an X-ray film.

### Treatment with prazosin, an α1-AR antagonist, rauwolscine, an α2-AR antagonist, and propranolol, a β-AR antagonist for membrane recruitment of TG2 and increase in GD3 synthase gene expression in K562 cells

Prazosin hydrochloride, rauwolscine hydrochloride, and propranolol hydrochloride (Sigma, #P8688) were purchased from Sigma–Aldrich. The drugs were appropriately dissolved in RPMI-1640 medium and stored at −20°C refrigerator until use. The drug was diluted in culture medium to prepare the required concentrations before use. K562 cells were treated with 10 μM epinephrine with or without 15 μM prazosin, 2 μM rauwolscine, and 15 μM propranolol. Then, GD3 synthase gene expression and membrane-bound TG2 levels were examined by RT-PCR and immunoblot analysis.

### Assay of erythroid differentiation

Levels of erythroid lineage differentiation of K562 cells was scored by erythroid-specific dye, benzidine staining method, as the procedure has been reported by Cooper *et al.* [58]. Benzidine dye-positive cells (blue) were visualized by phase contrast light microscopy.

### GTP binding assay

To detect photoaffinity labeling on cells, membrane proteins were isolated from K562 cells and incubated with 0.1 mCi of isotope [α-^32^P]GTP with 2 mM MgCl_2_ at RT for 20 min, transferred to a cold ice bath, and cross-linked for 8 min with UV irradiation [59]. GTP-bound TG2 was then immunoprecipitated with anti-TG2 antibody as described above. The radio-labeled GTP levels were visualized by autoradiography after electrophoretic separation of proteins using SDS-PAGE (10% gel).

### Measurement of TGase activity

As assay of TGase activity has previously been described [60], TGase activity was measured with 1% N,N’-dimethylcasein, 1 μM putrescine (2.2 × 10^6^ cpm/1 μM, final), and 300 μM Ca^2+^ at 25°C for 20 min.

### Electroporation and reporter luciferase assay

For transcriptional capacity of the GD3 synthase gene promoter, K562 cells was transiently transfected with each promoter-reporter by electroporation. In brief, the cultured cells were washed with PBS buffer (136 mM NaCl, 2 mM KCl, 0.9 mM Na_2_HPO_4_, and 1.7 mM KH_2_PO_4_, pH 7.4) containing each 10 μg of luciferase reporter construct. Five μg of a cytomegalovirus-β-galactosidase vector (pCMVβ) was separately used as a transfection efficiency control. The 0.5 ml sample containing approximately 2.2 × 10^6^ cells was suspended in PBS buffer and moved to a cuvette. Electroporation was performed using a Bio-Rad Gene Pulser II at 950 μF and 250 V, as recommended for usage. After electroporation, the cells were resuspended in RPMI 1640 medium containing 10% FBS and cultured for 12 h. Epinephrine (10 μM) was added to the cells for 12 h after transfection and the cells were further cultured for 24 h in order to induce erythroid differentiation. When the cells were harvested, the luciferase activities of promoter constructs were assayed using Luminoskan Ascent (Thermo Labsystems, Helsinki, Finland) and the dual-luciferase reporter assay system kit (Promega, Madison, WI). Luciferase activity was normalized to β-galactosidase activity.

### Electrophoretic mobility shift assay (EMSA)

Nuclear extracts from untreated and epinephrine-treated K562 cells were prepared as described [61]. A gel shift assay system kit (Promega, Madison, WI) has been used for EMSA, as recommended by the manufacturer's mannuals. Double-stranded oligonucleotides contained the following consensus sequences: AP-1 (5′-CGCTTGATGAGTCAGCCGGAA-3′), CREB (5′-AGAGATTGCCTGACGTCAGAGAGCTAG-3′), NF-κB (5′-AGTTGAGGGGACTTTCCCAGGC-3′), and SP-1 (5′-ATTCGATCGGGGCGGGGCGAGC-3′).

### Preparation and transfection of small interfering RNAs

Small interfering RNA (siRNA) duplexes were designed to target the coding sequences of human TGase and GD3 synthase mRNAs. They were synthesized and supplied by Bioneer Corp. (Daejon, Korea). The target coding sequences of TG2 siRNA and GD3 synthase siRNA were 5′-TGACCTAACCACTTAGCAT-3′ and 5′-GTGGCCGTCAAATAGATGA-3′, respectively. K562 cells were transfected with siRNA (10 μM) using Lipofectamine 2000 (Invitrogen, Carlsbad, CA) according to the manufacturer's manual. Twenty four hours after transfection, transfection complexes were removed and the culture medium was replaced with fresh medium. After incubation for 48 h, the siRNA transfected cells were used for experimental analysis.

### Data analysis and statistics

Data are presented as mean ± sd. Data were also statistically compared using the Student's *t*-test. A value of *P* < 0.05 was considered statistically significant.

## Abbreviations

AR: adrenergic receptor
CML: chronic myelogenous leukemia
CREB: cyclic-AMP response element binding protein
FBS: fetal bovine serum
PMA: phorbol 12-myristate 13-acetate
RT-PCR: reverse transcriptase-polymerase chain reaction
SDS-PAGE: sodium dodecyl sulfate-polyacrylamide gel electrophoresis
TG2: transglutaminase 2
